# Rational improvement of the engineered isobutanol-producing *Bacillus subtilis* by elementary mode analysis

**DOI:** 10.1186/1475-2859-11-101

**Published:** 2012-08-03

**Authors:** Shanshan Li, Di Huang, Yong Li, Jianping Wen, Xiaoqiang Jia

**Affiliations:** 1 Department of Biological Engineering, School of Chemical Engineering and Technology, Tianjin University, Tianjin, 300072, China; 2 Key Laboratory of Systems Bioengineering, Ministry of Education, Tianjin, 300072, China

**Keywords:** Rational strain improvement, Metabolic network, Elementary mode analysis, Target prediction, *Bacillus subtilis*, Isobutanol

## Abstract

**Background:**

Isobutanol is considered as a leading candidate for the replacement of current fossil fuels, and expected to be produced biotechnologically. Owing to the valuable features, *Bacillus subtilis* has been engineered as an isobutanol producer, whereas it needs to be further optimized for more efficient production. Since elementary mode analysis (EMA) is a powerful tool for systematical analysis of metabolic network structures and cell metabolism, it might be of great importance in the rational strain improvement.

**Results:**

Metabolic network of the isobutanol-producing *B. subtilis* BSUL03 was first constructed for EMA. Considering the actual cellular physiological state, 239 elementary modes (EMs) were screened from total 11,342 EMs for potential target prediction. On this basis, lactate dehydrogenase (LDH) and pyruvate dehydrogenase complex (PDHC) were predicted as the most promising inactivation candidates according to flux flexibility analysis and intracellular flux distribution simulation. Then, the *in silico* designed mutants were experimentally constructed. The maximal isobutanol yield of the LDH- and PDHC-deficient strain BSUL05 reached 61% of the theoretical value to 0.36 ± 0.02 C-mol isobutanol/C-mol glucose, which was 2.3-fold of BSUL03. Moreover, this mutant produced approximately 70 % more isobutanol to the maximal titer of 5.5 ± 0.3 g/L in fed-batch fermentations.

**Conclusions:**

EMA was employed as a guiding tool to direct rational improvement of the engineered isobutanol-producing *B. subtilis*. The consistency between model prediction and experimental results demonstrates the rationality and accuracy of this EMA-based approach for target identification. This network-based rational strain improvement strategy could serve as a promising concept to engineer efficient *B. subtilis* hosts for isobutanol, as well as other valuable products.

## Background

Isobutanol is considered as a leading candidate for the replacement of current fossil fuels [1,2]. Due to global environmental problems and fuel crises, isobutanol is expected to be produced in biotechnological process, which fulfills the demands of green and sustainable energy production [3]. Atsumi et al. [2] launched isobutanol bio-production in engineered *Escherichia coli* by harnessing the power of natural L-valine biosynthetic pathways. At present, isobutanol can be biosynthesized in several engineered microorganisms [2,4-8].

As the best-characterized Gram-positive microorganism, *Bacillus subtilis* is regarded as a promising isobutanol producer owing to some valuable features. In addition to high isobutanol toxicity tolerance, *B. subtilis* has no significant codon usage bias, which facilitates the functional heterologous gene expression and pathway engineering. Besides, it can secrete several enzymes to depolymerize polysaccharides that are presented in large amounts in plant, and further utilize some resulted oligosaccharides and C5 sugar (e.g. L-arabinose) [9], which benefits isobutanol production from low-value feedstocks. So far, *B. subtilis* has been engineered for isobutanol production [6], whereas it still needs to be improved for higher yield.

Pathway modifications that direct metabolic flux towards the desired products play an important role in strain optimization. Several corresponding metabolic strategies, such as pathway reconstruction [10] and cofactor manipulation [11], have been well applied for metabolic evolution of the isobutanol producers. Nevertheless, these approaches are always time-consuming and subjected to laborious experiments for target validation. As cells are elaborate systems with highly interconnected metabolic networks, it is challenging to capture the full range of behaviors of a cell and identify the accurate targets for efficient strain improvement by analyzing a set of linear pathways.

To solve this problem, it is necessary to investigate the cell behaviors systematically. Current state-of-the-art omics technologies together with the next-generation sequencing promote the progress of systems biology, which allows the quantitative understanding of pathway operations during cellular metabolism by using the mutually related mathematical modeling and experiment [12]. This kind of framework addresses the questions of traditional metabolic engineering, accelerates the strain improvement process, and opens the door to a new era of network-based strain evolution [13-17]. Presently, several computational tools are developed for systematic cellular metabolism analysis [18]. Among all of them, elementary mode analysis (EMA) is acknowledged as a powerful tool to identify the metabolic network properties. Based on the nullspace and convex analysis, as well as the steady-state, EMA decomposes the complex metabolic network of a cell into a set of unique and indivisible pathways, which link all possible cellular physiological states [19,20]. Knowledge of these pathways allows the rational *in silico* design of an ideal host with specialized metabolic functionalities. In addition to previous applications for theoretical yield analysis and cellular phenotype prediction, EMA has drawn more and more attentions to develop efficient bioconversion platforms for the desired chemicals [13,15,21,22].

EMA is an attractive approach for strain improvement, whereas few attempts were performed in the isobutanol-producing microorganisms except for the recent cases implemented by Trinh et al. [23] and Matsuda et al. [24]. Furthermore, EMA has not been employed to explore the metabolic behaviors of *B. subtilis* until now. Based on the preceding successes, here we presented a network-based EMA strategy to rationally improve the engineered isobutanol-producing *B. subtilis*. First, the genome-scale metabolic network model of this strain was reconstructed and refined. Then, potential targets that influence isobutanol biosynthesis were identified, and the strain engineering strategy was proposed. Finally, the *in silico* designed isobutanol-producing *B. subtilis* mutants were experimentally constructed and further tested to verify the model prediction.

## Results

### Metabolic network analysis of the isobutanol-producing *B. subtilis*

**Figure 1:**
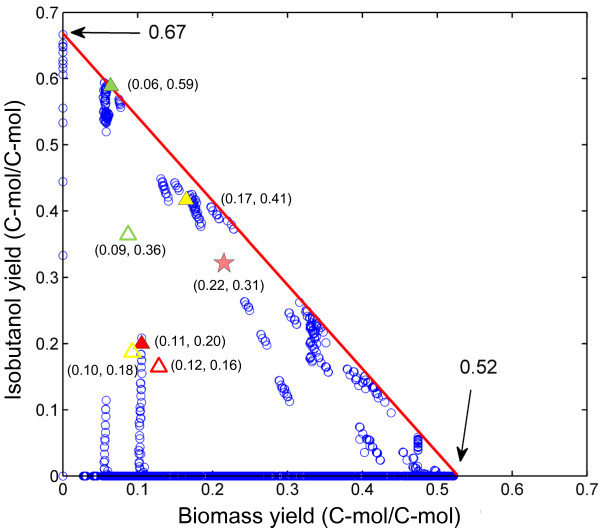
**EMs for isobutanol and biomass biosynthesis in isobutanol-producing *B. subtilis *BSUL03.** The solution of the EMs is represented by the blue circles. All possible solutions locate the interior as well as the sides of the rectangular triangle. EMs on the axes represent extreme modes exclusively linked to the formation of either isobutanol or biomass. The solid triangles indicate the best modes of the simulated mutants at optimal performance. The hollow triangles indicate the experimental values of different mutants in batch fermentations. Red, BSUL03; yellow, BSUΔ*ldh* or BSUL04; green, BSUΔ*ldh*Δ*pdhC* or BSUL05. The pink pentagram indicates the experimental values of BSUL05 in fed-batch fermentations.

Metabolic network of the isobutanol-producing *B. subtilis* BSUL03 for EMA comprises 131 reactions (36 reversible and 95 irreversible) and 132 metabolites (Additional file 1, Table S1 and Table S2). Overall, this metabolic network was decomposed into a total of 11,342 elementary modes (EMs). Each mode represents a unique possible pathway with balanced metabolites and cofactors. Among all the EMs, the majority are extreme modes exclusively linked to the production of either biomass or isobutanol, locating on the two axes of the plot (Figure 1). About 0.2% of the total EMs (25 EMs) allowed the maximal theoretical isobutanol yield of 0.67 C-mol isobutanol/C-mol glucose (C-mol/C-mol), all of them had no biomass and byproduct. Conversely, only EMs without isobutanol biosynthesis could reach the maximal biomass yield of 0.52 C-mol biomass/C-mol glucose, close to the value of the wild type *B. subtilis*[25]. Considering the coupled formation of isobutanol and biomass, the total EMs were constrained to 2,216 EMs (less than 20% of the total EMs) with the maximal theoretical isobutanol yield of 0.64 C-mol/C-mol. The large number of EMs illustrates the robustness and flexibility of the cells to adapt themselves to particular conditions by using different pathways.

### Potential target identification

EMA calculates the reaction flux and gives an extensive and profound insight into cell behaviors based on the metabolic network. Thus the potential bottlenecks could be investigated according to the flux distribution of EMs. For a precise prediction, EMs were classified and screened reasonably by taking the actual cellular physiological state into consideration. First, 2,216 EMs were generated by eliminating the extreme EMs from the total 11,342 EMs. Then, the unreasonable EMs (see Methods) were further excluded form 2,216 EMs, which finally resulted in 239 qualified EMs for the following analysis.

Statistical analysis was subsequently carried out. Flux correlation between each reaction and the objective reaction (R130) was scanned *in silico*. Reactions with valid flux correlation (linear regression coefficient *R*^2^ ≥ 0.7) and statistical significance (by *t*-test) were selected for flux flexibility analysis, which reflects the influence of the given reaction on the objective reaction [22]. Here, 53 eligible reactions were obtained for flux flexibility analysis. For a given reaction, flux flexibility was analyzed by calculating the flux distribution among the qualified EMs and evaluated by the coefficient of standard deviation (Vσ). Here, the top 12 candidates ranked via Vσ are listed in Table 1, including pyruvate branches (R20, R22 and R43), 2-ketoisovalerate (KIV) biosynthetic reactions (R59-R61), reactions involved in pentose phosphate pathway (PPP) (R31-R33, R36, R37) and one reaction responsible for redox balancing (R53). As shown in Table 1, reactions catalyzed by lactate dehydrogenase (LDH) and pyruvate dehydrogenase complex (PDHC) exhibited much higher flux flexibility than others. It suggests that the flux redistribution of the carbon drained off pyruvate is of great importance for isobutanol biosynthesis. Since both R43 and R22 showed the negative slope of linear regression with R130, LDH and PDHC were preliminarily selected as inactivation candidates for in vivo implementation in BSUL03.

**Table 1:** Potential targets predicted by EMA based on flux correlation

**No.**	**Enzyme**	**EC No.**	**Gene**	**Vσ**
R43	lactate dehydrogenase	EC-1.1.1.27	*ldh*	2.76
R22	pyruvate dehydrogenase complex	EC-1.2.1.51	*pdhABCD*	1.65
R59	acetolactate synthase	EC-4.1.3.18	*alsS*	0.70
R60	ketol-acid reductoisomerase	EC-1.1.1.86	*ilvC*	0.70
R61	dihydroxy-acid dehydratase	EC-4.2.1.9	*ilvD*	0.70
R53	transhydrogenase	-	-	0.57
R36	transketolase	EC-2.2.1.1	*tkt*	0.45
R37	transaldolase	EC-2.2.1.2	*ywjH*	0.44
R31	glucose 6-phosphate dehydrogenase	EC-1.1.1.49	*zwf*	0.39
R32	6-phosphogluconolactonase	EC-3.1.1.31	*ykgB*	0.39
R33	phosphogluconate dehydrogenase	EC-1.1.1.44	*yqjI*	0.39
R20	pyruvate kinase	EC-2.7.1.40	*pyk*	0.35

Detailed reactions are listed in Additional file 1, Table S1**.**

**Figure 2:**
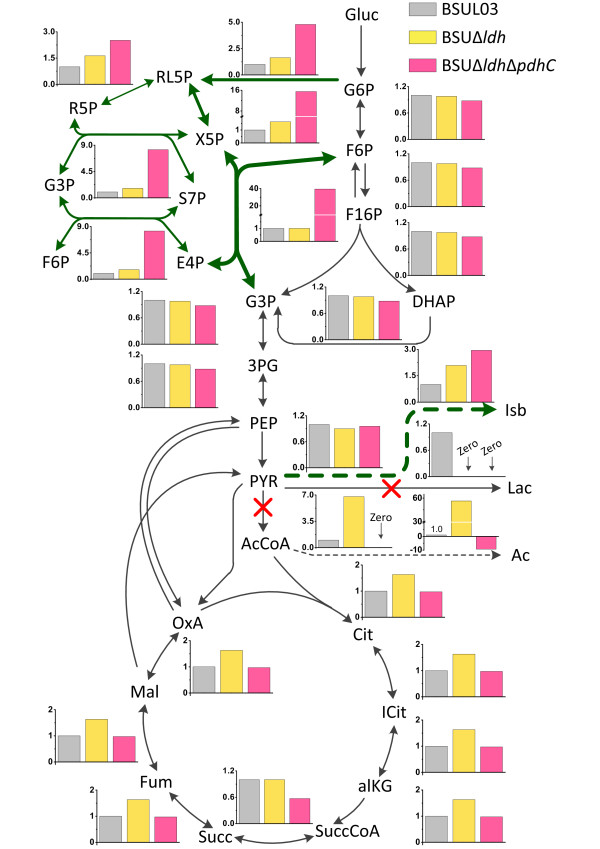
**Simulated flux-fold changes of the central metabolism of isobutanol-producing *B. subtilis *under the optimal conditions.** The chart represents the simulated fold change of the average flux through the central metabolism under optimal conditions considering the physiological states of different isobutanol-producing *B. subtilis*. All fluxes are given as relative molar flux normalized to 1 mol of glucose. Fold change of each reaction in the mutants is calculated by comparing to the corresponding flux in BSUL03. The dot lines indicate the multiple steps; the olive lines indicate the obviously increased flux by inactivating the targets (represented by red ×); the negative flux indicates a reaction occurs in reverse direction. Metabolites abbreviations: Gluc, Glucose; G6P, D-Glucose 6-phosphate; F6P, D-Fructose 6-phosphate; F16P, D-Fructose 1,6-bisphosphate; DHAP, Dihydroxyacetone phosphate; G3P, Glyceraldehyde 3-phosphate; 3PG, 3-Phospho-D-glycerate; PEP, Phosphoenolpyruvate; PYR, Pyruvate; AcCoA, Acetyl-coenzyme A; Cit, Citrate; ICit, Isocitrate; alKG, 2-Oxoglutarate; SuccCoA, Succinyl-CoA; Succ, Succinate; Fum, Fumarate; Mal, L-Malate; OxA, Oxaloacetate; RL5P, Ribulose-5-phosphate; R5P, alpha-D-Ribose 5-phosphate; X5P, Xylulose-5-phosphate; G3P, Glyceraldehyde 3-phosphate; S7P, Sedoheptulose 7-phosphate; E4P, D-Erythrose 4-phosphate; Isb, Isobutanol; Lac, L-Lactate; Ac, Acetate.

To ensure the validity of the chosen targets, intracellular flux distributions of the parental strain BSUL03 and its corresponding mutants were further simulated. When considering lactate production, the theoretical isobutanol yield of BSUL03 sharply decreased from 0.64 to 0.20 C-mol/C-mol under the optimal conditions (Figure 1). Pyruvate node analysis showed that pyruvate flux split ratio of LDH to acetolactate synthease (ALS) was 3.7:1, much higher than the values of LDH to the other branches. As the effect of gene knockout could be simulated by removing the corresponding reactions from the stoichiometric matrix, the phenotype of the specific mutant could be analyzed by the remaining EMs. As shown in Figure 2, the relative flux of the suppositional LDH-deficient strain BSUΔ*ldh* was obviously changed in tricarboxylic acid (TCA) cycle, PPP and isobutanol biosynthetic pathway under the optimal conditions. Compared to BSUL03, the twofold relative flux through ALS of BSUΔ*ldh* increased the theoretical isobutanol yield to 0.41 C-mol/C-mol (Figure 1). Additionally, pyruvate node analysis also showed that the flux drained off pyruvate was remarkably redistributed among the remaining branches. In particular, the flux fraction through pyruvate dehydrogenase complex (PDHC) was significantly increased by 10-fold (4.0% → 44.3%), higher than the 1.5-fold increase through ALS (19.9% → 49.4%). Synchronously, acetate secretion was enhanced by the excessive carbon flux through PDHC (Figure 2). In the suppositional LDH- and PDHC-deficient strain BSUΔ*ldh*Δ*pdhC*, ALS occupied 94% of pyruvate flux and became the dominant pyruvate gainer at optimal performance according to pyruvate node analysis. The relative flux through the objective reaction was approximately tripled in comparison with that of BSUL03 (Figure 2). As a result, this *in silico* strain obtained a 2-fold increase of the theoretical isobutanol yield of 0.59 C-mol/C-mol (Figure 1).

As stated above, inactivation of LDH and PDHC could obviously redirect the pyruvate flux towards isobutanol biosynthetic pathway, and thus improve the theoretical yield. Therefore, LDH and PDHC were finally chosen for in vivo implementation.

### Experimental validation—I. Construction and characterization of the LDH-deficient isobutanol-producing *B. subtilis*

According to the prediction, gene *ldh* encoding for LDH was disrupted in BSUL03 by integrating plasmid pUCLKm (Additional file 2, Figure S1A) into the chromosome of BSUL03. Recombinants resistant to kanamycin were selected for PCR confirmation by using a pair of primers *ldh*-F and *ldh*-R. BSUL03 showed a 0.9 kb PCR band, while the *ldh*-disrupted strain BSUL04 showed a 2 kb PCR band (Additional file 2, Figure S1B). Assay of enzyme activity showed that LDH activity of BSUL04 was 0.06 ± 0.01 U/mg, while that was 5.20 ± 0.06 U/mg of BSUL03. These results indicated that LDH activity was destroyed in BSUL04.

**Figure 3:**
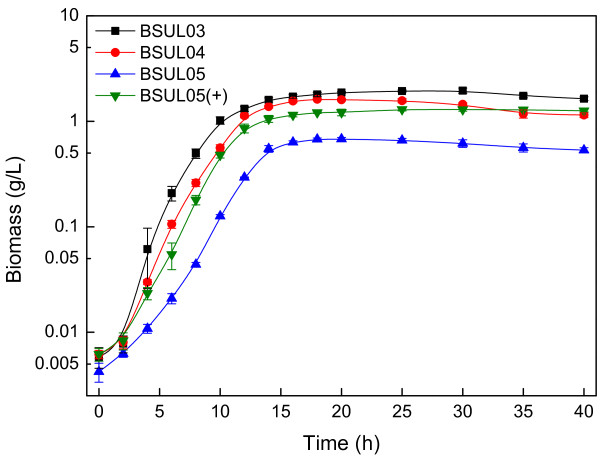
**Cell growth comparison of different isobutanol-producing *B. subtilis. *** The experiments were carried out in LBGSM-I medium under microaerobic conditions. The plus symbol indicates that the strain was cultivated in the medium supplemented with 3 g/L sodium acetic acid.

**Figure 4:**
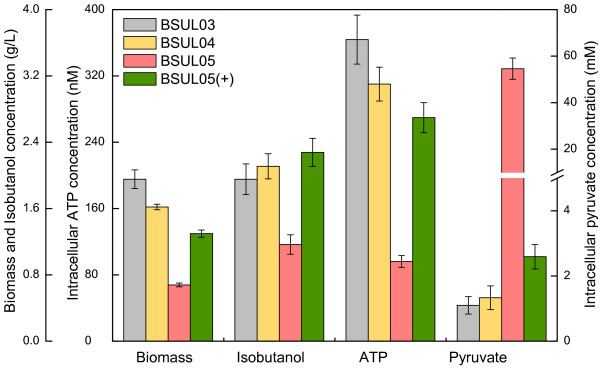
**Relationships of cell growth, isobutanol production, intracellular ATP and pyruvate of different isobutanol-producing *B. subtilis. *** The experiments were carried out in LBGSM-I medium under microaerobic conditions. The plus symbol indicates that the strain was cultivated in the medium supplemented with 3 g/L sodium acetic acid.

Strain BSUL04 showed a 21% and 25% lower biomass (1.62 ± 0.03 g/L) and specific growth rate (0.29 ± 0.01 h^-1^) than BSUL03 (Figure 3), respectively, suggesting that cell growth was impaired by *ldh* disruption. In microaerobic batch fermentations, BSUL04 produced 2.11 ± 0.15 g/L isobutanol with a yield of 0.18 ± 0.02 C-mol/C-mol, which was 12.5% higher than that of BSUL03. As expected, lactate was undetected in BSUL04, however, ethanol increased by 67% to 1.82 ± 0.27 g/L and acetate even tripled to 10.65 ± 1.04 g/L (Table 2). Combining with the almost unchanged intracellular pyruvate pool (Figure 4), these phenomena implied that the carbon flux originally consumed by LDH was mainly splitted by the competing branches via PDHC and isobutanol biosynthetic pathway via ALS in BSUL04, in agreement with pyruvate node analysis.

**Table 2:** **Comparison of metabolic profiles of different isobutanol-producing* B. subtilis* under microaerobic conditions**

**Strain**	**Isobutanol production**	**Isobutanol yield**	**Lactate**	**Acetate**	**Ethanol**
	**(g/L)**	**(C-mol/C-mol)**	**(g/L)**	**(g/L)**	**(g/L)**
BSUL03	1.95 ± 0.18	0.16 ± 0.01	3.67 ± 022	3.73 ± 0.29	1.09 ± 0.13
BSUL04	2.11 ± 0.15	0.18 ± 0.02	ND	10.65 ± 1.04	1.82 ± 0.27
BSUL05	1.17 ± 0.12	0.36 ± 0.02	ND	ND	ND
BSUL05(+)	2.28 ± 0.17	0.29 ± 0.01	ND	4.46 ± 0.85	ND

Strains were cultured in LBGSM-I medium for 40 h under microaerobic conditions. The plus symbol indicates medium for BSUL05 cultivation is supplemented with 3 g/L sodium acetic acid; ND represents the product is not detected.

### Experimental validation—II. Construction and characterization of the LDH- and PDHC-deficient isobutanol-producing *B. subtilis*

In *B. subtilis*, the nonpolar PDHC-deficient mutants can be obtained by the interruptions of any gene involved in *pdhABCD* operon except for the essential gene *pdhA*[26]. As the core of PDHC, the E2 subunits encoded by *pdhC* affect PDHC activity most significantly. Therefore, *pdhC* was reasonably selected for gene knockout in the present work. Recombinants with kanamycin and tetracycline resistance suggested that *pdhC* were deleted by integrating the plasmid pUCPTet (Additional file 2, Figure S1C) into the chromosome of BSUL04 via double homologous recombinant. By using a pair of primers *pdhC*1-F and *pdhC*2-R, the band of BSUL04 and the *pdhC*-disrupted *B. subtilis* BSUL05 were 2 and 2.75 kb, respectively (Additional file 2, Figure S1D). PDHC activity of BSUL03 and BSUL04 were 0.31 ± 0.01 U/mg and 0.27 ± 0.01 U/mg, respectively, whereas it could not be detected in BSUL05, implying that PDHC was inactivated in BSUL05.

Strain BSUL05 exhibited a longer transition time to exponential phase: 4 h compared to 2 h in BSUL03 and BSUL04. Besides, biomass (0.68 ± 0.02 g/L) and the specific growth rate (0.17 ± 0.02 h^-1^) were 35 % and 44 % of BSUL03, respectively (Figure 3). Along with the suppressed cell growth, isobutanol production and intracellular ATP concentration of BSUL05 sharply decreased to 1.17 ± 0.12 g/L and 96 ± 7 nM, respectively. Meanwhile, a conspicuous increase of intracellular pyruvate concentration (approximately by 50-fold) was also observed (Figure 4). Satisfactorily, lactate, acetate and ethanol were undetected in fermentation broth (Table 2). In comparison with BSUL04, BSUL05 doubled isobutanol yield to 0.36 ± 0.02 C-mol/C-mol, which was 61% of the predicted value (0.59 C-mol/C-mol). Though cell growth and isobutanol production were inhibited in BSUL05, both of them could be well restored by external acetate addition (Table 2). Simultaneously, the intracellular ATP concentration increased by 180% to 270 ± 18 nM, and the intracellular pyruvate concentration decreased by 95% to 2.6 mM (Figure 4). However, an unexpected net acetate accumulation was noticed during fermentations (Table 2).

### Isobutanol biosynthesis profile of *B. subtilis* BSUL05

**Figure 5:**
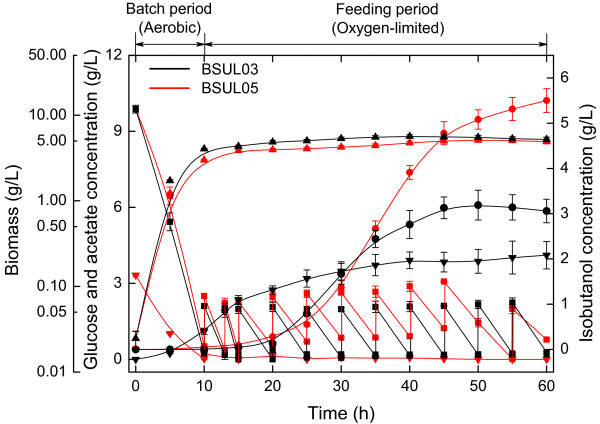
**Comparison of metabolic profiles of BSUL03 and BSUL05 in fed-batch fermentations.** Time-course profiles of cell growth, isobutanol, glucose and acetate in fed-batch fermentations were compared between BSUL05 and the parental strain BSUL03. Symbols: square, glucose; circle, isobutanol; triangle, biomass; downtriangle, acetate.

Strain BSUL05 showed an improved isobutanol biosynthetic capability in batch fermentations. Here it was further assessed in fed-batch fermentations. At the same time, the parental strain BSUL03 was taken as control. As shown in Figure 5, both strains grew exponentially under the aerobic conditions. Though glucose and acetate were almost exhausted during this period, little isobutanol was detected in the fermentation broth. After entering the oxygen-limited period, cell growth of the two strains slowed down, whereas isobutanol biosynthesis began to accelerate. Despite of the almost identical cell growth tendency, the two strains presented different isobutanol biosynthetic behaviors. Isobutanol titer of BSUL03 reached the peak (3.2 ± 0.4 g/L) around 50 h and declined thereafter, whereas that of BSUL05 continuously increased to 5.5 ± 0.3 g/L at the end of fermentations. Meanwhile, isobutanol yield was up to 53% of the theoretical value to 0.31 ± 0.02 C-mol/C-mol, which was 1.9-fold of BSUL03. Contrastively, the final acetate concentration of BSUL05 decreased to 0.2 g/L, which was merely about 10% of that of BSUL03. These results demonstrate the better isobutanol biosynthetic performance of the rational improved BSUL05 than the parental strain.

## Discussion

Some progress is being made in strain improvement of the engineered isobutanol producers [2,4,6]. However, these strategies are always time-consuming and laborious due to the incomplete understanding of the complex cellular behaviors. Network-based EMA is believed to be an attractive approach to handle this problem, while the relevant investigations are rather limited in isobutanol producers. For that reason, the EMA-based design strategy was first employed as a guiding tool to tailor the engineered *B. subtilis* for better isobutanol-producing performance [6].

EMA decomposes a metabolic network into a set of unique and non-divisible pathways that represent all possible physiological states of the cells. Different from algorithms such as MOMA [27] and OptGene [28] that could identify only one optimal solution, EMA calculates all the possible optimal pathway solutions. It offers an opportunity to investigate the flux distribution at different performances, comprehend the underlying cellular behaviors, and draft the blueprint for strain improvement.

For a precise prediction, the qualified EMs should be chosen from the total EMs, as only those conformed to the real cellular physiological state are meaningful to strain optimization. Here, two points should be taken into account. One is that the extreme EMs without the synchronous formation of isobutanol and biomass need to be eliminated. The other one is that the central metabolism is active under the oxygen-limited condition despite the expression of the involved genes are downregulated [11,29]. Therefore, EMs with non-positive carbon flux through glycolysis and the irreversible reactions of PPP and TCA cycle are unreasonable and also need to be excluded. Previous reports showed that linear relationship existed between fluxes through the target candidates and the objective reaction [22,30]. Therefore, the statistically significant reactions were picked out for further analysis. Both flux flexibility analysis (Table 1) and intracellular flux simulation (Figure 2) proposed LDH and PDHC as the most promising inactivation targets. This fully coincides with the fact that both LDH (*K*_*m*_ = 3.0 mM) [31] and PDHC (*K*_*m*_ = 4.3 mM) [32] possess much higher pyruvate affinity than ALS (*K*_*m*_ = 13.6 mM) [33] in *B. subtilis*. In addition, LDH can be activated in an FNR (a transcriptional activator for anaerobically induced genes) independent manner under oxygen-limited conditions [29]. Following *ldh* and *pdh*C, *alsSilvC* and *ilvD* involved in KIV biosynthetic reactions were identified as potential amplification targets (Table 1), holding an identical view with the previous findings that overexpression of these genes is beneficial to isobutanol production enhancement [2,5,6]. Apart from the above targets, potential candidates also included a transhydrogenase and five PPP-related encoding genes, implying that the intracellular redox state maintains close ties with isobutanol biosynthesis.

As analyzed above, the corresponding mutants were experimentally constructed to validate the EMA prediction. Disruption of *ldh* eliminated lactate and increased isobutanol yield by 12.5 %, meaning that the carbon flux is indeed redirected towards isobutanol as simulated. Although the observations were consistent with the findings from several experimental studies [4,5], the yield increment was not so much as predicted. We speculated that this could be primarily put down to the undesirably drastic increase of acetate, which is a well-known inhibitor in fermentation. Romero et al. [34] demonstrated that, in *B. subtilis*, LDH plays a pivotal role in maintaining the redox equilibrium (NAD(P)H/NAD(P)^+^) under fermentative conditions. As ketol-acid reductoisomerase and alcohol dehydrogenase for isobutanol biosynthesis require NADPH and NADH as cofactor, respectively, the discrepancy might also be ascribed to the depressed enzyme activity, which was relevant to the disturbed intracellular redox state caused by *ldh* disruption. This conjecture agreed with the speculation about the intimate connection between cellular redox state and isobutanol biosynthesis during target identification. Further disruption of *pdhC* obviously doubled isobutanol yield to 0.36 ± 0.02 C-mol/C-mol, and decreased acetate and ethanol to an undetectable level (Table 2). These data suggested that, on one aspect, PDHC inactivation triggered a significant carbon flux shift, which favors isobutanol biosynthesis and was confirmed by intracellular metabolites analysis (unpublished data). On another aspect, byproduct elimination prevented the broth from overacidification and increased isobutanol yield [35]. Therefore, EMA is accurate enough to predict the targets and guide the rational improvement of the engineered isobutanol-producing *B. subtilis*.

When taking glucose as the sole carbon source, both cell growth and isobutanol production were suppressed in BSUL05 (Figure 3 and Table 2). As shown in Figure 1, EMA reveals an inverse relationship between the yield of isobutanol and biomass owing to the shared precursor competition [15,22]. Thus, it was plausible to observe that strain with higher isobutanol biosynthetic efficiency exhibited a lower cell growth. Furthermore, the impaired cell growth of this PDHC-deficient strain might also be accounted for the lack of a pivotal building block, acetyl-coenzyme A (AcCoA). As for the decreased isobutanol production, it might be explained by two reasons. On one hand, an appropriate biomass is necessary for the production of desired products, so the lower isobutanol production of BSUL05 might be attributed to the impaired cell growth. On the other hand, the depressed TCA cycle caused by AcCoA shortage led to ATP scarcity (Figure 4), which influenced cell growth and normal expression of the enzymes for isobutanol biosynthesis. Delightedly, we found that external acetate addition could increase the intracellular ATP concentration and restore the impaired cell growth along with isobutanol production (Figure 4). These results turned out that acetate could not only serve as a carbon source, but also as an energy source in BSUL05. Unexpectedly, a net acetate accumulation was observed, which might be relevant to the putative pyruvate oxidase (POX) in *B. subtilis*[36]. Originally, the small amount of acetate biosynthesized via POX could be assimilated via acetyl-CoA synthetase, whereas this kind of balance might be destroyed by the accelerated metabolism induced by external acetate addition. Fortunately, acetate could be well controlled at a low concentration in fed-batch fermentations (Figure 5), implying the acetate production-consumption equilibrium could be balanced under appropriate conditions.

The rational improved isobutanol-producing *B. subtilis* BSUL05 displayed much better isobutanol biosynthetic performances than the parental strain BSUL03 both in batch and fed-batch fermentations (Table 2 and Figure 5). However, the maximal isobutanol yield of BSUL05 obtained in the present work (0.36 ± 0.02 C-mol/C-mol) was 39 % lower than the *in silico* prediction (0.59 C-mol/C-mol) (Figure 1). Such a discrepancy was also noticed by other researchers [15]. It is inferred that this phenomenon might be explained by the following reasons. First, EMA revealed that efficient isobutanol biosynthesis depends on the low oxygen level (data not shown), whereas the precise control of the oxygen-limited condition is really a challenge [4,23]. Then, as EMA hinted the important role of cellular redox state in isobutanol biosynthesis (Table 1 and Figure 2), this discrepancy might partially ascribe to the disturbed redox equilibrium caused by *ldh* and *pdhC* deletion [4,5]. Next, isobutanol biosynthesis might be inhibited by some negative effects of the cell regulatory system that was not considered by EMA. Besides, some unknown regulation mechanisms could also interpret the fact. Finally, as the ‘just-in-time’ gene transcription (and the associated enzyme expression) shows clear influence on efficient bio-production [37,38], the lower isobutanol yield of the mutant might be attributed to the uncoordinated isobutanol biosynthetic pipelines, which could not be explored by the metabolic network. Therefore, the aforementioned factors need to be investigated in future studies to construct a more efficient isobutanol-producing *B. subtilis*. At these points, the improved performance of BSUL05 has confirmed that EMA is a valuable tool for rapid and precise target identification to engineer *B. subtilis* with stronger isobutanol biosynthetic capability. This metabolic network-based rational strain improvement strategy could be applied to construct valuable industrial *B. subtilis* hosts for the production of isobutanol and other desired products.

## Conclusions

In this work, we presented the first report on rational improvement of the isobutanol-producing *B. subtilis* by employing an EMA-based strategy. Flux flexibility analysis and *in silico* simulation predicted LDH and PDHC as the most promising inactivation targets, which were further validated experimentally. The maximal isobutanol yield and titer of the mutant BSUL05 reached 2.3- and 1.7-fold of BSUL03 to 0.36 ± 0.02 C-mol/C-mol and 5.5 ± 0.3 g/L, respectively, showing a stronger isobutanol biosynthetic capability. The consistency between model prediction and experimental results demonstrates that EMA is a reliable approach for target identification and strain optimization. Moreover, EMA dropped a hint of the close relationship between isobutanol biosynthesis and cellular redox state, which provides a valuable insight into further improvement of the isobutanol-producing *B. subtilis*. Our results demonstrate that the EMA-based prediction could serve as a useful strategy to guide strain engineers towards improved bio-production in *B. subtilis*, as well as other microorganisms.

## Methods

### Metabolic network reconstruction

The genome-scale metabolic network of isobutanol-producing *B. subtilis* was constructed by introducing the isobutanol biosynthetic reactions, which allows the conversion from KIV to isobutanol, into the previously described *B. subtilis* 168 network [39]. For EMA, the model was further refined. Except for the central carbon metabolism, linear reactions involved in other subsystems were lumped when necessary (Additional file 1, Table S1). It takes glucose as the sole carbon source via phosphotransferase system, and contains other substrates ammonium, sulfur and oxygen. Products of isobutanol, lactate, acetate, ethanol, valine, carbon dioxide and biomass, as well as ATP are considered as external metabolites. For ATP production in the respiratory chain, a P/O ratio of 1.33 (for NADH) and 0.89 (for FADH_2_) was assumed [40]. The biomass composition and cell molecular were taken from Dauner and Sauer [41]. Water, protons and phosphate were assumed to be ubiquitous and unlimited in the cells.

### EMA

EMA was implemented by METATOOL [42]. The script files and compiled shared library of METATOOL 5.1 were downloaded from the METATOOL website (http://www.biozentrum.uni-wuerzburg.de/bioinformatik/). EMA results were analyzed using Excel Microsoft Corp. for mode sorting and filtering.

### Potential target identification based on flux correlation

For a given elementary flux mode *j*, the relative metabolic flux (${\text{v}}_{i,j}$) of each reaction *i* is normalized to the glucose uptake flux. The yield (${\text{Y}}_{P/C,\text{j}}$) of biomass and isobutanol was calculated according to Eq. 1.

(1) $${\text{Y}}_{\text{P/C,j}}\text{=}\frac{{\text{S}}_{\text{p,j}}}{{\text{S}}_{\text{C,j}}}\frac{{\text{ξ}}_{\text{p,j}}}{{\text{ξ}}_{\text{C,j}}}\text{, }1\leq \text{j}\leq \text{n}$$

Here, S stands for the EMs matrix with the dimension of *j* × *i*., where *i* and *j* are the number of reactions and EMs, respectively. The symbol ξ represents the molar carbon content expressed in C-mol per mol biomass or isobutanol.

To investigate whether a reaction *i* is a potential target, a chosen set of EMs was screened for further statistical analysis by considering the cellular physiological state of isobutanol biosynthesis. First, the extreme EMs were excluded from the total EMs. Then, EMs with non-positive carbon flux through glycolysis and the irreversible reactions of PPP and TCA cycle were unreasonable and further eliminated, generating the qualified EMs for statistical analysis. The linear flux correlation between the reaction *i* and R130 was primarily analyzed. Here, a cut-off value of the regression coefficient *R*^2^ = 0.7 was set for preliminary target selection [22]. If the linear slope is positive, the reaction *i* is regarded as the amplification target, otherwise it is the gene knockout candidate. Besides, the statistical significance of these targets was also examined by *t*-test, which was a criterion to classify the qualified reactions for flux flexibility analysis. Flux flexibility reflects the influence of a given reaction *i* on the objective reaction. It is analyzed by calculating the flux distribution among the qualified EMs and measured by Vσ as described in Eq. 2. Thus, the potential target could be ranked via the value of Vσ.

(2) $${\text{Vσ}}_{i}=\frac{\delta_{v_{i}}}{v_{i}},\;1\leq \text{i}\leq \text{m}$$

Here, $\delta_{v_{i}}$ and $\bar{v_{i}}$ refer to the deviation and the average flux of reaction *i*, respectively. The variable *m* refers to the number of reactions in the metabolic network.

### Microbial strains and media

All strains and plasmids used in this work are listed in Table 3. All Bacillus mutants used in this work were derivatives of BSUL03 [6]. *E. coli* JM109 was used to propagate all plasmids. Unless stated otherwise, *E. coli* and *B. subtilis* were cultured in Luria-Bertani (LB) medium (peptone 10 g/L, yeast extract 5 g/L, and sodium chloride 5 g/L) at 37 °C. Batch fermentations were carried out in LBGSM-I medium (LB medium supplemented with glucose 20 g/L, K_2_HPO_4_ 2 g/L, KH_2_PO_4_ 1 g/L and 10^3^ dilution of Trace Metal Mix A5 [2]). Fed-batch fermentations were carried out in LBGSM-III medium (identical to LBGSM-I except for glucose 10 g/L). Glucose feeding solution (glucose 500 g/L) and acetate feeding solution (sodium acetic acid 200 g/L) were used as feeding stocks during fed-batch fermentations. Antibiotics were added appropriately as follows: ampicillin 100 μg/mL, spectinomycin 100 μg/mL, kanamycin 10 μg/mL and tetracycline 20 μg/mL.

**Table 3:** Strains and plasmids used in this study

**Name**	**Relevant genotype**	**Source**
**Strains**		
*E. coli* JM109	*recA*1, *endA*1, *gyrA*96, *thi*-1, *hsdR*17, *supE*44, *relA*1, Δ(*lac-proAB)*/*F*’[*traD*36, *proAB*+, *lacIq, lacZ*Δ*M*15]	TransGen Biotech
BSUL03	Δ*amyE*::(P_43_::*kivd*-*adh*2), P_43_::*ilvD*-*ilvC*-*alsS*; Spc^r^, Em^r^	[6]
BSUL04	BSUL03 with lactate dehydrogenase inactivation (Δ*ldh*); Spc^r^, Em^r^, Km^r^	This study
BSUL05	BSUL04 with pyruvate dehydrogenase complex E2 subunit inactivation (Δ*ldh*Δ*pdhC*); Spc^r^, Em^r^, Km^r^, Tet^r^	This study
**Plasmids**		
pUC18	*E. coli* cloning vector; Amp^r^	Laboratory stock
pDK	*B. subtilis* integration vector with *amyE* locus; Amp^r^, Km^r^	BGSC [50]
pHY300PLK	*E. coli*-*B. subtilis* cloning vector; Amp^r^, Tet^r^	Takara
pUCL	pUC18 containing *ldh* fragment of *B. subtilis*, Amp^r^	This study
pUCLKm	pUCL containing kanamycin resistance cassettes from pDK; Amp^r^, Km^r^	This study
pUCP01	pUC18 containing homology arm *pdhC*1 fragment of *B. subtilis*; Amp^r^	This study
pUCP02	pUCP01 containing homology arm *pdhC*2 fragment of *B. subtilis*; Amp^r^	This study
pUCPTet	pUCP02 containing tetracycline resistance cassettes from pHY300PLK; Amp^r^, Tet^r^	This study

### Gene cloning and plasmid construction

All oligonucleotides used in this work are listed in (Additional file 3 Table S3). Standard techniques for nucleic acid manipulation were used as described by Sambrook et al. [43]. By using genomic DNA of *B. subtilis* BSUL03 as template, the *ldh* gene (code for LDH) [GenBank: 938348] was amplified with a pair of primers *ldh*-F and *ldh*-R, the homologous arms *pdhC*1 and *pdhC*2 of *pdhC* (code for PDHC E2 subunit) [GenBank: 936010] were amplified with two pairs of primers *pdhC*1-F and *pdhC*1-R, *pdhC*2-F and *pdhC*2-R, respectively. The *Xba*I-*Xma*I digested *ldh* PCR product was cloned into pUC18 cut with the same enzymes to create pUCL. Then the kanamycin-resistant cassette cut from plasmid pDK with *EcoR*I and *EcoR*V was blunted and ligated into the plasmid pUCL cut with *EcoR*V, creating pUCLKm (Additional file 2, Figure S1A). Plasmid pUCP01 was obtained by cloning the *Hind*III-*Pst*I digested *pdhC*1 PCR product into pUC18 cut with the same enzymes. Then the *BamH*I-*EcoR*I digested *pdhC*2 PCR product was cloned into pUCP01 cut with the same enzymes, creating pUCP02. The tetracycline-resistant cassette was amplified by using plasmid pHY300PLK as template with a pair of primers Tet-F and Tet-R. The *Pst*I-*BamH*I digested PCR product was then cloned into pUCP02 cut with the same enzymes, creating pUCPTet (Additional file 2, Figure S1C).

### Construction of isobutanol-producing *B. subtilis* mutants

To obtain *B. subtilis* mutants, the integration vectors pUCLKm and pUCPTet were sequentially transformed into BSUL03 cells by using the competent cell method [44]. *B. subtilis* recombinants were selected by kanamycin resistance or/and tetracycline resistance, and further confirmed by PCR using two pairs of primers *ldh*-F/*ldh*-R and *pdhC*1-F/*pdhC*2-R, respectively. The LDH mutant and the LDH and PDHC mutant were designated as BSUL04 and BSUL05, respectively.

### Cultivation

Pre-cultures were prepared by cultivating one fresh colony in the liquid LB medium at 240 rpm for 8 h. The 1% (v/v) inoculation was adopted in all experiments. Batch fermentations for phenotype growth and metabolic profile assays were carried out in 500 mL screw-cap flasks with LBGSM-I medium under the microaerobic conditions (40% work volume, 200 rpm, 37°C) for 40 h. Fed-batch fermentations were performed in 400 mL LBGSM-III cultures in a fed-batch Pro fermentation system (DASGIP, Germany) under two-stage (aerobic/oxygen-limited) conditions for 60 h. Dissolved oxygen was measured by an oxygen electrode (Mettler Toledo, Germany). For aerobic conditions (0–10 h), it was controlled at 30 ± 1% of saturation in a cascade by stirring from 200 to 700 rpm with 1 volume of air per volume of medium per minute (vvm). For oxygen-limited conditions (10–60 h), it was adjusted to 5 ± 1% by reducing the stirrer and aeration to 50 rpm and 0.5 vvm, respectively. The pH adjusted by 2 M NaOH and 2 M HCl was maintained at 7.0 by a standard pH electrode (Mettler Toledo, Germany). Foam was prohibited by manual injection of appropriate antifoamer (Sigma 204). When glucose concentration fell below 1 g/L, 1.6 mL of glucose feeding solution was added. For PDHC-deficient strains, sodium acetic acid were originally supplemented into the medium at a final concentration of 3 g/L and 3.4 g/L in batch and fed-batch fermentations, respectively. Besides, 0.4 mL of acetate feeding solution was added coupled with glucose feeding solution during feeding period. All the fermentative experiments were performed in triplicate.

### Enzyme activity assays

Cells for LDH and PDHC enzyme activity assays were grown at 37°C in 25 mL LB medium, harvested at the exponential phase (6 h) and the beginning of the stationary phase (14 h), respectively, washed twice with cold potassium phosphate buffer (100 mM, pH 7.0), and then suspended in 5 mL of the same buffer. Cell extracts were obtained by sonication (UH-250A, Autoscience instrument Co., Ltd.; 40 k Hz, 10-s pulse and 5-s intervals, total 10 min) and centrifugation (17,900 g for 10 min at 4°C). The supernatant was kept on ice until determination. The standard assay for LDH activity (towards lactate) was spectrophotometrically (340 nm) monitored at 25°C by following the oxidation of NADH [45]. The activity of the PDHC was performed as described by Murarka et al. [46]. Total protein concentrations were measured by Bradford assay [47].

### Measurement of intracellular ATP concentration

The intracellular ATP concentration was measured by the ATP assay kit (Beyotime, China). Cells (1 × 10^4^) harvested at the mid-log phase were mixed with 20 μL lysis buffer and homogenized by vortex, and then centrifuged at 15,300 g for 5 min at 4°C. Other procedures such as lysis buffer preparation, background luminescence correction and ATP measurement were performed as the ATP Assay protocol (Beyotime). The emitted light by luminescent reaction was quantified by a luminometer (Synergy H4 Hybrid Microplate reader, BioTek, USA). ATP concentrations of the samples were calculated from the standard curve using linear regression (0.5-50 nM). Each experiment was carried out in triplicate and each sample was measured five times.

### Intracellular pyruvate analysis

To analyze the intracellular pyruvate, 5 mL mid-log phase cell suspension was harvested using vacuum filtration (AP-01P Vacuum Pump, Tianjin Auto Science Co., Ltd.; cellulose nitrate, 0.22 μm pore size) and washed three times each with 20 mL 0.9 % cold NaCl solution (the whole filtration procedure including the washing was finished in less than 30 s) [48]. Metabolite extraction and sample preparation were carried out as described [49]. Sampling was carried out five times in parallel for each experiment.

Intracellular pyruvate analysis was implemented by liquid chromatography-tandem quadrupole mass spectrometry (Bruker MicroTOF-Q II, Germany) equipped with a 250 × 4.6 mm aminopropyl column (Luna NH_2_, 5 μm particle size, Phenomenex). The detailed separation and elution conditions, as well as data analysis approaches were carried out as described previously [49].

### Analytical methods

Cell concentration was determined by measuring the optical density of culture broth at 600 nm (OD_600_). Biomass was calculated by multiplying OD_600_ by a conversion factor of 0.325. The quantitative analysis of glucose and fermentation metabolites (alcohols, organic acids and other compounds) were also performed as before [6].

## Abbreviations

EMA: elementary mode analysis; EMs: elementary modes; LDH: lactate dehydrogenase; PDHC: pyruvate dehydrogenase complex; ALS: acetolactate synthease; KIV: 2-ketoisovalerate; POX: pyruvate oxidase; AcCoA: acetyl-coenzyme A; EMP: glycolysis pathway; PPP: pentose phosphate pathway; TCA cycle: tricarboxylic acid cycle; LB: Luria-Bertani.

## Competing interests

The authors declare that they have no competing interests.

## Authors’ contributions

JPW and SSL conceived and designed the research. SSL performed the main experiments, the statistical analysis and drafted the manuscript. DH participated in model simulation and manuscript revision. YL performed mutant construction and enzyme assays. XQJ contributed to metabolites analysis. JPW supervised the research and revised the manuscript. All authors read and approved the final manuscript.

## Supplementary Material

Additional file 1 **Metabolic network model of the engineered isobutanol-producing *B. subtilis *BSUL03.** Details of model construction are described in Additional file 1. Stoichiometric equations involved in the model for EMA are listed in Table S1. All abbreviations of metabolites are listed in Table S2. Click here for file

Additional file 2 **Plasmids construction and gene knockout confirmation.** Plasmids used for gene disruption of *ldh* (A) and *pdhC* (C). PCR confirmation of gene knockout for *ldh* (B) and *pdhC* (D). The genomic DNA of BSUL03 was used as control. Lane M, 1 kb DNA ladder; Lane S, the positive double crossover mutant; Lane +, the positive control; Lane -, the negative control. Click here for file

Additional file 3 Oligonucleotides used in this study. Click here for file
