# Supplementary material for: Rational improvement of the engineered isobutanol-producing Bacillus subtilis by elementary mode analysis
Source: Microb Cell Fact. 2012 Aug 3;11:101. doi: 10.1186/1475-2859-11-101 (PMC3475101; doi:10.1186/1475-2859-11-101)
Supplement: Additional file 1 — Metabolic network model of the engineered isobutanol-producing B. subtilis BSUL03. Details of model construction are described in Additional file 1. Stoichiometric equations involved in the model for EMA are listed in Table S1. All abbreviations of metabolites are listed in Table S2. [file 1475-2859-11-101-S1.pdf]

Additional File 1 Model reconstruction

The metabolic reactions of the metabolic model of *Bacillus subtilis* for isobutanol production were derived from the recently published genome-scale metabolic network [1, 2]. The metabolic network of isobutanol-producing *B. subtilis* used for metabolic pathway analysis was constructed as follows. (i) Isobutanol biosynthetic reaction (R130) was introduced in the *B. subtilis* stoichiometric model to covert α-ketoisovalerate into isobutanol. (ii) The original genome-scale metabolic network was refined to take glucose as the primary substrate via phosphotransferase system (PTS). (iii) For ATP production in the respiratory chain a P/O ratio of 1.33 (for NADH) and 0.89 (for $FADH_2$) was assumed [3]. (iv) The biomass composition and cell molecular (Mr=25.03 g/mol, D=0.1 h^{-1}) were taken from Dauner and Sauer [4]. (v) Except for the central carbon metabolism, the linear reactions involved in other metabolic subsystems were lumped when necessary. (vi) It is assumed that water, protons and phosphate are ubiquitous in the cells and not limiting.

Table S1 Stoichiometric equations of the metabolic model of isobutanol-producing *B. subtilis* BSUL03

In the reaction equations, GDP, CMP, UDP and UMP were omitted for simplicity [5]. Except for biomass with the unit of “mg”, other units of stoichiometric coefficient are in “mol”. Symbol ‘-->’ indicates irreversible reactions, ‘<==>’ indicates reversible reactions.

Subsystem	No.	Reaction	Genes
Transport reactions	R1	1 Gluc_ext + 1 PEP --> 1 G6P + 1 Pyr	(*ptsH* or *ptsG* or *ptsI*)
	R2	1 O2_ext --> 1 O2	
	R3	1 N_ext --> 1 N	*nrgA*
	R4	1 ATP + 4 NADPH + 1 S_ext --> 1 AMP + 1 S + 4 NADP	(*ybaR* or *cysP* or *yvdB*)
	R5	1 CO2 <==> 1 CO2_ext	
	R6	1 Lac --> 1 Lac_ext	(*yvfH* or *lctP*)
	R7	1 Eth --> 1 Eth_ext	
	R8	1 Ac --> 1 Ac_ext	
	R9	1 Isb --> 1 Isb_ext	
	R10	1 Val --> 1 Val_ext	
	R11	1 ATP --> 1 ATP_ext	
Glycolysis	R12	1 G6P <==> 1 F6P	*pgi*
	R13	1 F6P + 1 ATP --> 1 F16P + 1 ADP	*pfkA*
	R14	1 F16P <==> 1 DHAP + 1 G3P	(*fbaA* or *fbaB*)
	R15	1 DHAP <==> 1 G3P	*tpiA*
	R16	1 G3P + 1 NAD <==> 1 DPG + 1 NADH	(*gapA* or *gapB*)
	R17	1 DPG + 1 ADP <==> 1 3PG + 1 ATP	*pgk*
	R18	1 3PG <==> 1 2PG	*pgm*
	R19	1 2PG <==> 1 PEP	*eno*
	R20	1 PEP + 1 ADP --> 1 Pyr + 1 ATP	*pyk*
	R21	1 F16P --> 1 F6P	*fbp*
TCA cycle	R22	1 Pyr + 1 CoA + 1 NAD --> 1 AcCoA + 1 NADH + 1 CO2	(*pdhD* or (*pdhA* and *pdhB*) or *pdhC*)
	R23	1 AcCoA + 1 OxA --> 1 Cit + 1 CoA	(*citA* or *citZ*)
	R24	1 Cit <==> 1 ICit	*citB*
	R25	1 ICit + 1 NADP <==> 1 alKG + 1 NADPH + 1 CO2	*icd*

	R26	1 alKG + 1 CoA + 1 NAD --> 1 SuccCoA + 1 NADH + 1 CO2	(*pdhD* or *odhB* or *odhA*)
	R27	1 SuccCoA + 1 ADP <==> 1 Succ + 1 ATP + 1 CoA	(*sucC* or *sucD*)
	R28	1 Succ + 1 FAD <==> 1 Fum + 1 $FADH_2$	(*sdhB* and *sdhA* and *sdhC*)
	R29	1 Fum <==> 1 Mal	*citG*
	R30	1 Mal + 1 NAD <==> 1 OxA + 1 NADH	*mdh*
PP Pathway	R31	1 G6P + 1 NADP --> 1 PGlac + 1 NADPH	*zwf*
	R32	1 PGlac --> 1 PGluc	*ykgB*
	R33	1 PGluc + 1 NADP --> 1 RL5P + 1 NADPH + 1 CO2	*yqjI*
	R34	1 RL5P <==> 1 X5P	*ywlF*
	R35	1 RL5P <==> 1 R5P	*rpe*
	R36	1 R5P + 1 X5P <==> 1 G3P + 1 S7P	*tkt*
	R37	1 G3P + 1 S7P <==> 1 F6P + 1 E4P	*ywjH*
	R38	1 E4P + 1 X5P <==> 1 F6P + 1 G3P	*tkt*
Pyruvate branches	R39	1 AcCoA + 1 NADH <==> 1 Acald + 1 NAD + 1 CoA	
	R40	1 NADH + 1 Acald <==> 1 Eth + 1 NAD	
	R41	1 AcCoA <==> 1 AcP + 1 CoA	*pta*
	R42	1 AcP + 1 ADP <==> 1 ATP + 1 Ac	*ackA*
	R43	1 Pyr + 1 NADH <==> 1 Lac + 1 NAD	*ldh*
	R44	2 ATP + 1 Ac + 1 CoA --> 1 AcCoA + 1 AMP	*acsA*
Anaplerotic reactions	R45	1 ATP + 1 Pyr + 1 CO2 --> 1 OxA + 1 ADP	*pycA*
	R46	1 OxA + 1 ATP --> 1 PEP + 1 CO2 + 1 ADP	*pckA*
	R47	1 PEP + 1 CO2 + 1 ADP --> 1 OxA + 1 ATP	*pckA*
	R48	1 Mal + 1 NADP --> 1 Pyr + 1 NADPH + 1 CO2	*ytsJ*
Coenzymes and Energy	R49	1 ATP + 1 NADPH + 1 THF <==> 1 MLTHF + 1 NADP + 1 ADP	
	R50	1 NADH + 1 FAD <==> 1 $FADH_2$ + 1 NAD	(*yjlD* or *yutJ* or *yumB*)
	R51	1 NADH + 0.5 O2 --> 1.33 ATP + 1 NAD	((*ctaF* and *ctaE* and *ctaD* and *ctaC*) or (*qcrC* and *qcrB*

			and *qcrA*))
	R52	1 $FADH_2$ + 0.5 O2 --> 0.89 ATP + 1 FAD	(*sdhB* and *sdhA* and *sdhC*)
	R53	1 NADH + 1 NADP <==> 1 NADPH + 1 NAD	*yqjI*
	R54	1 ATP <==> 1 ADP	
Amino acid biosynthesis	R55	2 PEP + 1 E4P + 1 ATP + 1 NADPH --> 1 Chor + 1 NADP + 1 ADP	(*aroA* or *aroB* or *aroC* or *aroD* or *aroK* or *aroE* or *aroF*)
	R56	1 R5P + 1 ATP <==> 1 PRPP + 1 AMP	*prs*
	R57	2 ATP + 1 Gln + 1 CO2 --> 1 CBP + 1 Glu + 2 ADP	((*carA* and *carB*) or (*pyrAB* and *pyrAA*))
	R58	1 Pyr + 1 Glu --> 1 alKG + 1 Ala	*alaT*
	R59	2 Pyr --> 1 Alac + 1 CO2	*alsS*
	R60	1 Alac + 1 NADPH <==> 1 DHIV + 1 NADP	(*ilvH* and *ilvB*)
	R61	1 DHIV <==> 1 3Mob	(*ilvC* or *ilvD*)
	R62	1 3Mob + 1 Glu <==> 1 alKG + 1 Val	(*ywaA* or *ybgE*)
	R63	1 3Mob + 1 AcCoA + 1 NAD --> 1 NADH + 1 CO2 + 1 4Mop + 1 CoA	(*leuA* or (*leuC* and *leuD*) or *leuB*)
	R64	1 4Mop + 1 Glu <==> 1 alKG + 1 Leu	(*ybgE or ywaA*)
	R65	1 ATP + 1 Gln + 1 Asp --> 1 Asn + 1 Glu + 1 AMP	(*asnB* or *asnH* or *asnO*)
	R66	1 OxA + 1 Glu --> 1 alKG + 1 Asp	*aspB*
	R67	1 Asp --> 1 Fum + 1 N	*ansB*
	R68	1 ATP + 1 NADPH + 1 Asp --> 1 AspSAld + 1 NADP + 1 ADP	(*yclM* or *dapG* or *lysC* or *asd*)
	R69	1 NADPH + 1 AspSAld --> 1 Hser + 1 NADP	*hom*
	R70	1 26dap_M --> 1 CO2 + 1 Lys	*lysA*
	R71	1 SuccCoA + 1 MLTHF + 1 HSer + 1 Cys + 1 NADPH --> 1 Pyr + 1 Succ + 1 N + 1 Met + 1 CoA + 1 THF	(*metA* or *yjcI* or *metE* or *yitJ*)

	R72	1 ATP + 1 HSer --> 1 Thr + 1 ADP	(*thrB* or *thrC*)
	R73	1 Pyr + 1 NADPH + 1 Thr --> 1 CO2 + 1 N + 1 3Mop + 1 NADP	(*ilvA* or (*ilvH* and *ilvB*) or *alsS* or *ilvC* or *ilvD*)
	R74	1 3Mop + 1 Glu <==> 1 alKG + 1 Ile	(*ywaA* or *ybgE*)
	R75	1 ATP + 1 PRPP + 1 Gln + 2 NAD --> 1 AICAR + 1 alKG + 2 NADH + 1 His	(*hisG* or *hisI* or *hisA* or *hisB* or *hisF* or *hisH* or *hisC* or *hisD* or *hisJ*)
	R76	1 alKG + 1 NADPH + 1 N --> 1 Glu + 1 NADP	(*yrpC* or *racE*)
	R77	1 ATP + 1 N + 1 Glu --> 1 Gln + 1 ADP	*glnA*
	R78	1 ATP + 2 NADPH + 1 Glu --> 1 Pro + 1 ADP + 2 NADP	(*proB* or *proJ* or *proA* or *proG* or *proI* or *proH*)
	R79	1 Asp + 2 ATP + 1 CBP + 1 AcCoA + 2 Glu + 1 NADPH --> 1 Ac + 1 Arg + 1 Fum + 1 alKG + 1 AMP	(*argJ* or *argB* or *argC* or *argD* or *ylmB* or *argF* or *argG* or *argH*)
	R80	1 Chor + 1 PRPP + 1 Gln + 1 Ser --> 1 G3P + 1 Pyr + 1 CO2 + 1 Glu + 1 Trp	((*trpE* and *pabA*) or *trpD* or *trpF* or t*rpC* or (*trpA* and *trpB*))
	R81	1 Chor + 1 Glu + 1 NAD --> 1 alKG + 1 NADH + 1 CO2 + 1 Tyr	(*pheB* or *aroH* or *aroA* or *tyrA* or *hisC* or *hisH*)
	R82	1 Chor + 1 Glu --> 1 alKG + 1 CO2 + 1 Phe	(*pheB* or *aroH* or *aroA* or *pheA* or *hisC* or *hisH*)
	R83	1 3PG + 1 Glu + 1 NAD --> 1 alKG + 1 NADH + 1 Ser	(*serA* or *serC* or *rsbX*)
	R84	1 Ser + 1 THF <==> 1 MLTHF + 1 Gly	*glyA*
	R85	1 AcCoA + 1 S + 1 Ser --> 1 Ac + 1 Cys + 1 CoA	(*cysE* or *cysK*)
Nucleotide biosynthesis	R86	5 ATP + 2 Gln + 1 MLTHF + 1 Gly + 1 PRPP + 1 CO2 + 1 Asp --> 2 Glu + 5 ADP + 1 AICAR + 1 Fum + 1 THF	(*purF* or *purD* or *purN* or *purS* or *purL* or *purQ* or *purM* or *purK* or *purE* or *purC* or

			purB)
	R87	1 MLTHF + 1 AICAR <==> 1 Fprica	*purH*
	R88	1 Fprica --> 1 IMP	*purH*
	R89	1 Asp + 1 rGTP + 1 IMP --> 1 rATP + 1 Fum + 1 GDP	(*purA* or *purB*)
	R90	3 ATP + 1 Gln + 1 IMP + 1 NAD --> 1 NADH + 1 Glu + 1 rGTP + 1 AMP + 2 ADP	(*guaB* or *guaA* or *gmk* or *ndk*)
	R91	1 ATP + 1 N + 1 rUTP --> 1 Rctp + 1 ADP	*pyrG*
	R92	2 ATP + 1 Asp + 1 CBP + 0.5 O_2 + 1 PRPP --> 1 CO_2 + 1 rUTP + 1 CO_2 + 2 ADP	(*pyrB* or *pyrC* or (*pyrD* and *pyrK*) or *pyrF* or *katA* or *katE* or *katX* or *pyrH* or *cmk* or *ndk*)
	R93	1 NADPH + 1 rATP --> 1 dATP + 1 NADP	((*nrdE* and *nrdF* and *ymaA*) or *ndk*)
	R94	1 NADPH + 1 rGTP --> 1 dGTP + 1 NADP	((*nrdE* and *nrdF* and *ymaA*) or *ndk*)
	R95	1 NADPH + 1 rCTP --> 1 dCTP + 1 NADP	((*nrdE* and *nrdF* and *ymaA*) or *ndk*)
	R96	2 NADPH + 1 MLTHF + 1 rUTP --> 1 dTTP + 1 THF + 2 NADP	((*nrdE* and *nrdF* and *ymaA*) or *ndk* or *yncF* or *yosS* or *thyB* or *thyA* or *tmk* or *ndk)*
Lipid Biosynthesis	R97	1 G6P + 1 rUTP --> 1 UDPGlc	(*ybbT* or *gtaB*)
	R98	1 DHAP + 1 NADH --> 1 Glyc3P + 1 NAD	*gpsA*
	R99	6 ATP + 1 3Mob + 5 AcCoA + 10 NADPH + 1 NAD --> 1 fa1CoA + 1 NADH + 5 ADP + 4 CoA + 1 AMP + 10 NADP	(*ywpB* or *fabF* or *fabG* or *fabL* or *fabHB* or *fabD* or *fabI* or *fabF* or *fabG* or *ycsD* or *fabHA* or *fabD*)
	R100	7 ATP + 7 AcCoA + 12 NADPH --> 1 TdCoA + 6 ADP + 1 AMP + 12	(*fabF* or *fabI* or *fabG* or *ycsD*

	NADP + 6 CoA	or *fabHA* or *fabD* or *ywpB* or *fabF* or *fabG* or *fabL* or *fabHB* or *galE)*
R101	6 ATP + 1 4Mop + 5 AcCoA + 10 NADPH + 1 NAD --> 1 fa3CoA + 1 NADH + 4 CoA + 5 ADP + 1 AMP + 10 NADP	(*fabF* or *ywpB* or *fabG* or *fabL* or *fabHB* or *fabD* or *fabI* or *fabF* or *fabG* or *ycsD* or *fabHA* or *fabD*)
R102	6 ATP + 1 3Mop + 5 AcCoA + 10 NADPH + 1 NAD --> 1 fa4CoA + 1 NADH + 4 CoA + 5 ADP + 1 AMP + 10 NADP	(*fabF* or *ywpB* or *fabG* or *fabHB* or *fabL* or *fabD* or *fabF* or *fabI* or *fabG* or *ycsD* or *fabHA* or *fabD*)
R103	7 ATP + 1 3Mob + 6 AcCoA + 12 NADPH + 1 NAD --> 1 fa6CoA + 1 NADH + 5 CoA + 6 ADP + 1 AMP + 12 NADP	(*ywpB* or *fabF* or *fabG* or *fabHB* or *fabL* or *fabD* or *fabF* or *fabI* or *fabG* or *ycsD* or *fabHA* or *fabD*)
R104	8 ATP + 8 AcCoA + 14 NADPH --> 1 PmtCoA + 7 CoA + 14 NADP	(*ywpB* or *fabF* or *fabG* or *fabL* or *fabHB* or *fabD* or *fabI* or *fabF* or *fabG* or *ycsD* or *fabD*)
R105	7 ATP + 1 4Mop + 6 AcCoA + 12 NADPH + 1 NAD --> 1 fa11CoA + 1 NADH + 5 CoA + 6 ADP + 1 AMP + 12 NADP	(*fabF* or *fabI* or *fabG* or *ycsD* or *fabHA* or *fabD* or *fabF* or *ywpB* or *fabG* or *fabHB* or *fabL* or *fabD*)
R106	7 ATP + 1 3Mop + 6 AcCoA + 12 NADPH + 1 NAD --> 1 fa12CoA + 1 NADH + 5 CoA + 6 ADP + 1 AMP + 12 NADP	(*fabF* or *fabI* or *fabG* or *ycsD* or *fabD* or *fabF* or *ywpB* or *fabG* or *fabHB* or *fabL* or *fabD*)

	R107	9 ATP + 9 AcCoA + 16 NADPH --> 1 StrCoA + 16 NADP + 9 CoA	(*fabF* or *ywpB* or *fabG* or *fabL* or *fabHB* or *fabD* or *fabI* or *fabF* or *fabG* or *ycsD* or *fabHA* or *fabD*)
	R108	0.14 fa11CoA + 0.34 fa12CoA + 0.02 fa1CoA + 0.4 fa3CoA + 0.68 fa4CoA + 0.1 fa6CoA + 1 Glyc3P + 0.2 PmtCoA + 0.06 StrCoA + 0.06 TdCoA --> 0.01 12dag3p_BS	
	R109	0.01 12dag3p_BS + 1 rCTP --> 0.01 cdpdag_BS	*cdsA*
	R110	0.01 12dag3p_BS --> 0.01 12dgr_BS	
	R111	0.01 cdpdag_BS + 1 Glyc3P --> 0.01 pgly_BS	*pgsA*
	R112	0.01 cdpdag_BS + 1 Ser --> 1 CO2 + 0.01 psetha_BS	*pssA*
	R113	0.01 12dgr_BS + 1 UDPGlc --> 0.01 m12dg_BS	*ugtP*
	R114	0.01 12dgr_BS + 2 UDPGlc --> 0.01 d12dg_BS	*ugtP*
	R115	0.01 12dgr_BS + 3 UDPGlc --> 0.01 t12dg_BS	*ugtP*
	R116	1 ATP + 1 Lys + 0.01 pgly_BS --> 0.01 lysylpgly_BS + 1 AMP	*yfiX*
	R117	0.02 pgly_BS --> 0.01 cdlp_BS	(*ywiE* or *ywjE* or *ywnE*)
Cell wall Biosynthesis	R118	1 F6P + 1 AcCoA + 1 ATP + 1 Gln --> 1 Glu + 1 UDP_NAG + 1 CoA	(*gcaD* or *glmS* or *ybbT*)
	R119	1 Pyr + 1 SuccCoA + 1 NADPH + 1 AspSAld + 1 Glu --> 1 alKG + 1 Succ + 1 26dap_M + 1 CoA + 1 NADP	(*dapA* or *dapB* or *ykuQ* or *patA* or *ykuR* or *dapF*)
	R120	1 rCTP + 1 Glyc3P --> 1 CDPGLYC	*tagD*
	R121	45 CDPGLYC + 2 UDP_NAG --> 1 gtca1_45_BS	(*tagA* or *tagB* or *tagO* or *tagG* or *tagH* or *tagF*)
	R122	45 CDPGLYC + 2 UDP_NAG + 45 UDPGlc --> 1 gtca3_45_BS	(*tagA* or *tagB* or *tagE* or *tagO* or *tagG* or *tagH* or *tagF*)
	R123	45 Ala + 45 ATP + 45 CDPGLYC + 2 UDP_NAG --> 1 gtca2_45_BS	(*tagA* or *dltA* or *dltB* or *dltD* or *tagB* or *dltC* or *tagO* or

			tagG or t*agH* or *tagF*)
	R124	30 UDP_NAG + 30 UDPGlc --> 1 tcam_BS	(*ggaA* or *ggaB*)
	R125	2 UDP_NAG + 3 Ala + 5 ATP + 1 26dap_M + 1 Glu + 1 NADPH + 1 PEP --> 1 peptido_BS + 1 NADP + 5 ADP	
	R126	2400 CDPGLYC + 1 d12dg_BS --> 1 lipo4_24_BS	
	R127	2400 CDPGLYC + 1 d12dg_BS + 2400 UDPGlc --> 1 lipo1_24_BS	
	R128	2400 CDPGLYC + 1 d12dg_BS + 2400 UDP_NAG --> 1 lipo2_24_BS	
	R129	2400 Ala + 2400 ATP + 2400 CDPGLYC + 1 d12dg_BS --> 1 lipo3_24_BS + 2400 AMP	(*dltA* or *dltB* or *dltD* or *dltC*)
Isobutanol biosynthesis	R130	1 3Mob + 1 NADH --> 1 CO2 + 1 Isb + 1 NAD	(*kivd or adh2*)
Biomass	R131	105 ATP + 0.4083 Gly + 0.2669 Ala + 0.3067 Val + 0.3464 Leu + 0.2699 Ile + 0.2162 Ser + 0.1863 Thr + 0.1759 Phe + 0.1108 Tyr + 0.0543 Trp + 0.0567 Cys + 0.1133 Met + 0.3231 Lys + 0.193 Arg + 0.0817 His + 0.148 Asp + 0.2604 Glu + 0.148 Asn + 0.2603 Gln + 0.1606 Pro + 0.0505 rATP + 0.0627 rGTP + 0.0389 rCTP + 0.0415 rUTP + 0.0230 dATP + 0.0174 dGTP + 0.0174 dCTP + 0.0229 dTTP + 0.000085750 m12dg_BS + 0.000110292 d12dg_BS + 0.000065833 t12dg_BS + 0.000004642 cdlp_BS + 0.000175859 pgly_BS + 0.000022057 lysylpgly_BS + 0.000559509 psetha_BS + 0.000006837 lipo1_24_BS + 0.000006123 lipo2_24_BS + 0.000018162 lipo3_24_BS + 0.000014676 lipo4_24_BS + 0.10182 peptido_BS + 0.00362 gtca1_45_BS + 0.00235 gtca2_45_BS + 0.00182 gtca3_45_BS + 0.00311 tcam_BS + 0.0002 NADPH --> Biomass + 0.0002 NADP + 105 ADP	

Table S2 Abbreviations of metabolites

Abbreviation	Metabolite Name	Abbreviation	Metabolite Name
12dag3p_BS	1,2-diacyl-sn-glycerol 3-phosphate	gtca2_45_BS	glycerol teichoic acid (n=45), unlinked, D-ala substituted
12dgr_BS	1,2-diacylglycerol	gtca3_45_BS	glycerol teichoic acid (n=45), unlinked, glucose substituted
26dap_M	meso-2,6-Diaminoheptanedioate	His	L-Histidine
2PG	D-Glycerate 2-phosphate	Hser	L-Homoserine
3Mob	3-Methyl-2-oxobutanoate	ICit	Isocitrate
3Mop	(S)-3-Methyl-2-oxopentanoate	Ile	L-Isoleucine
3PG	3-Phospho-D-glycerate	IMP	IMP
4Mop	4-Methyl-2-oxopentanoate	Isb	isobutanol
Ac	Acetate	Isb_ext	isobutanol (extracellular)
Ac_ext	Acetate (extracellular)	Lac	L-Lactate
Acald	Acetaldehyde	Lac_ext	L-Lactate (extracellular)
AcCoA	Acetyl-CoA	Leu	L-Leucine
AcP	acyl carrier protein	lipo1_24_BS	lipoteichoic acid (n=24), linked, glucose substituted
ADP	ADP	lipo2_24_BS	lipoteichoic acid (n=24), linked, N-acetyl-D-glucosamine
AICAR	5-Amino-1-(5-Phospho-D-ribosyl)imidazole -4-carboxamide	lipo3_24_BS	lipoteichoic acid (n=24), linked, D-alanine substituted
Ala	L-Alanine	lipo4_24_BS	lipoteichoic acid (n=24), linked, unsubstituted
Alac	(S)-2-Acetolactate	Lys	L-Lysine
alKG	2-Oxoglutarate	lysylpgly_BS	lysylphophatidylglycerol
AMP	AMP	m12dg_BS	monoglucosyl-1,2 diacylglycerol
Arg	L-Arginine	Mal	L-Malate

Asn	L-Asparagine	Met	L-Methionine
Asp	L-Aspartate	MLTHF	5,10-Methylenetetrahydrofolate
AspSAld	L-Aspartate 4-semialdehyde	N	Ammonium
ATP	ATP	N_ext	Ammonium (extracellular)
ATP_ext	ATP (extracellular)	NAD	Nicotinamide adenine dinucleotide
CBP	Carbamoyl phosphate	NADH	Nicotinamide adenine dinucleotide - reduced
cdlp_BS	cardiolipin (*B. subtilis*)	NADPH	Nicotinamide adenine dinucleotide phosphate
cdpdag_BS	CDPdiacylglycerol (*B. subtilis*)	O2	O2
CDPGLYC	CDPglycerol	O2_ext	O2 (extracellular)
Chor	Chorismate	OxA	Oxaloacetate
Cit	Citrate	PEP	Phosphoenolpyruvate
CO2	CO2	peptido_BS	Peptidoglycan subunit of *Bacillus subtilis*
CO2_ext	CO2 (extracellular)	PGlac	6-phospho-D-glucono-1,5-lactone
CoA	Coenzyme A	PGluc	6-Phospho-D-gluconate
Cys	L-Cysteine	pgly_BS	phosphatidylglycerol (*B. subtils*)
d12dg_BS	diglucosyl-1,2 diacylglycerol	Phe	L-Phenylalanine
dATP	dATP	PmtCoA	Palmitoyl-CoA (n-C16:0 CoA)
dCTP	dCTP	Pro	L-Proline
dGTP	dGTP	PRPP	5-Phospho-alpha-D-ribose 1-diphosphate
DHAP	Dihydroxyacetone phosphate	psetha_BS	phosphatidylethanolamine (*B. subtilis*)
DHIV	(R)-2,3-Dihydroxy-3-methylbutanoate	Pyr	Pyruvate
DPG	3-Phospho-D-glyceroyl phosphate	R5P	alpha-D-Ribose 5-phosphate
dTTP	dTTP	rATP	ATP for RNA synthesis
E4P	D-Erythrose 4-phosphate	rCTP	CTP for RNA synthesis
Eth	Ethanol	rGTP	GTP for RNA synthesis
Eth_ext	Ethanol (extracellular)	RL5P	Ribulose-5-phosphate
F16P	D-Fructose 1,6-bisphosphate	rUTP	UTP for RNA synthesis

F6P	D-Fructose 6-phosphate	S	Sulfate
fa11CoA	Iso-C17:0 CoA	S_ext	Sulfate (extracellular)
fa12CoA	Anteiso-C17:0 CoA	S7P	Sedoheptulose 7-phosphate
fa1CoA	Iso-C14:0 CoA	Ser	L-Serine
fa3CoA	Iso-C15:0 CoA	StrCoA	Stearyl-CoA (n-C18:0 CoA)
fa4CoA	Anteiso-C15:0 CoA	Succ	Succinate
fa6CoA	Iso-C16:0 CoA	SuccCoA	Succinyl-CoA
FAD	FAD	t12dg_BS	triglucosyl-1,2 diacylglycerol
$FADH_2$	$FADH_2$	tcam_BS	minor teichoic acid (acetylgalactosamine glucose phosphate, n=30)
Fprica	5-Formamido-1-(5-phospho-D-ribosyl)imidazole-4-carboxamide	TdCoA	Tetradecanoyl-CoA (n-C14:0 CoA)
Fum	Fumaric acid	THF	5,6,7,8-Tetrahydrofolate
G3P	Glyceraldehyde 3-phosphate	Thr	L-Threonine
G6P	D-Glucose 6-phosphate	Trp	L-Tryptophan
GDP	GDP	Tyr	L-Tyrosine
Gln	L-Glutamine	UDP_NAG	UDP-N-acetylglucosamine
Gluc_ext	D-Glucose	UDPGlc	UDPglucose
Gly	Glycine	Val	L-Valine
Glyc3P	sn-Glycerol 3-phosphate	Val_ext	L-Valine (extracellular)
gtca1_45_BS	glycerol teichoic acid (n=45), unlinked, unsubstituted	X5P	Xylulose-5-phosphate

References

1. Henry C, Zinner J, Cohoon M, Stevens R: **iBsu1103: a new genome-scale metabolic model of *Bacillus subtilis* based on SEED annotations.** *Genome Biol* 2009, **10:**R69.
2. Oh Y-K, Palsson BO, Park SM, Schilling CH, Mahadevan R: **Genome-scale Reconstruction of Metabolic Network in *Bacillus subtilis* Based on High-throughput Phenotyping and Gene Essentiality Data.** *J Biol Chem* 2007, **282:**28791-28799.
3. Sauer U, Bailey JE: **Estimation of P-to-O ratio in *Bacillus subtilis* and its influence on maximum riboflavin yield.** *Biotechnol Bioeng* 1999, **64:**750-754.
4. Dauner M, Sauer U: **Stoichiometric growth model for riboflavin-producing *Bacillus subtilis*.** *Biotechnol Bioeng* 2001, **76:**132-143.
5. Stelling J, Klamt S, Bettenbrock K, Schuster S, Gilles ED: **Metabolic network structure determines key aspects of functionality and regulation.** *Nature* 2002, **420:**190-193.
