# Supplementary figures and images for: Rational improvement of the engineered isobutanol-producing Bacillus subtilis by elementary mode analysis
Source: Microb Cell Fact. 2012 Aug 3;11:101. doi: 10.1186/1475-2859-11-101 (PMC3475101; doi:10.1186/1475-2859-11-101)

**A**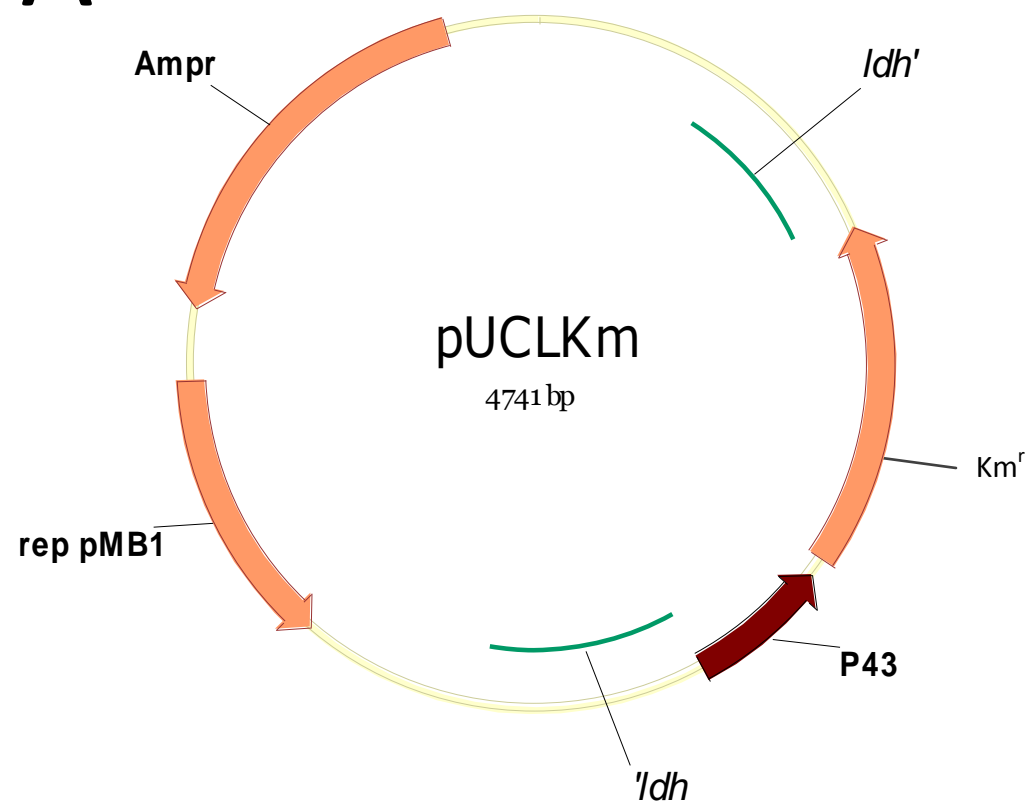**B**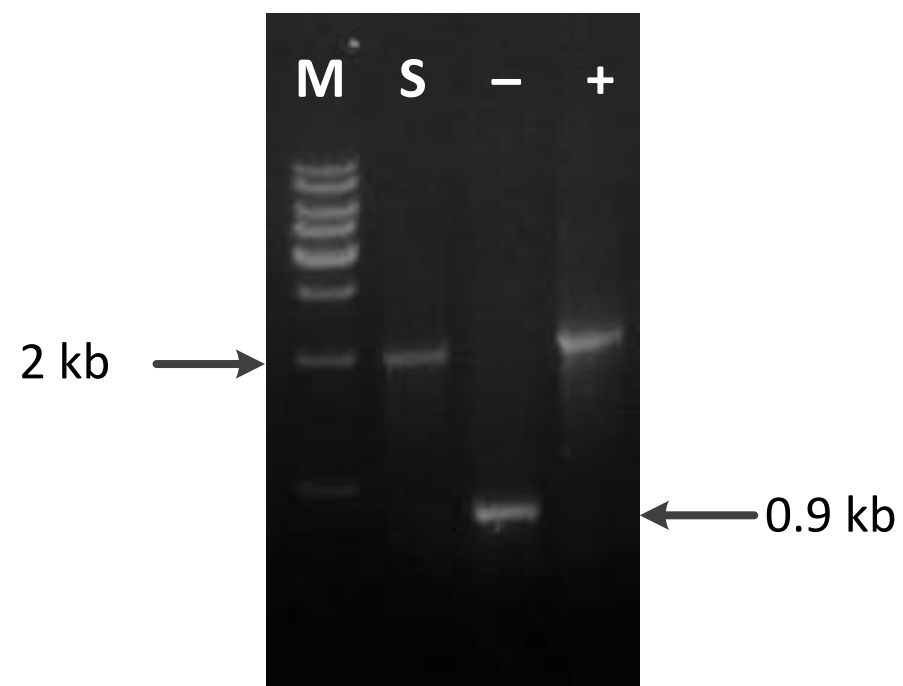**C**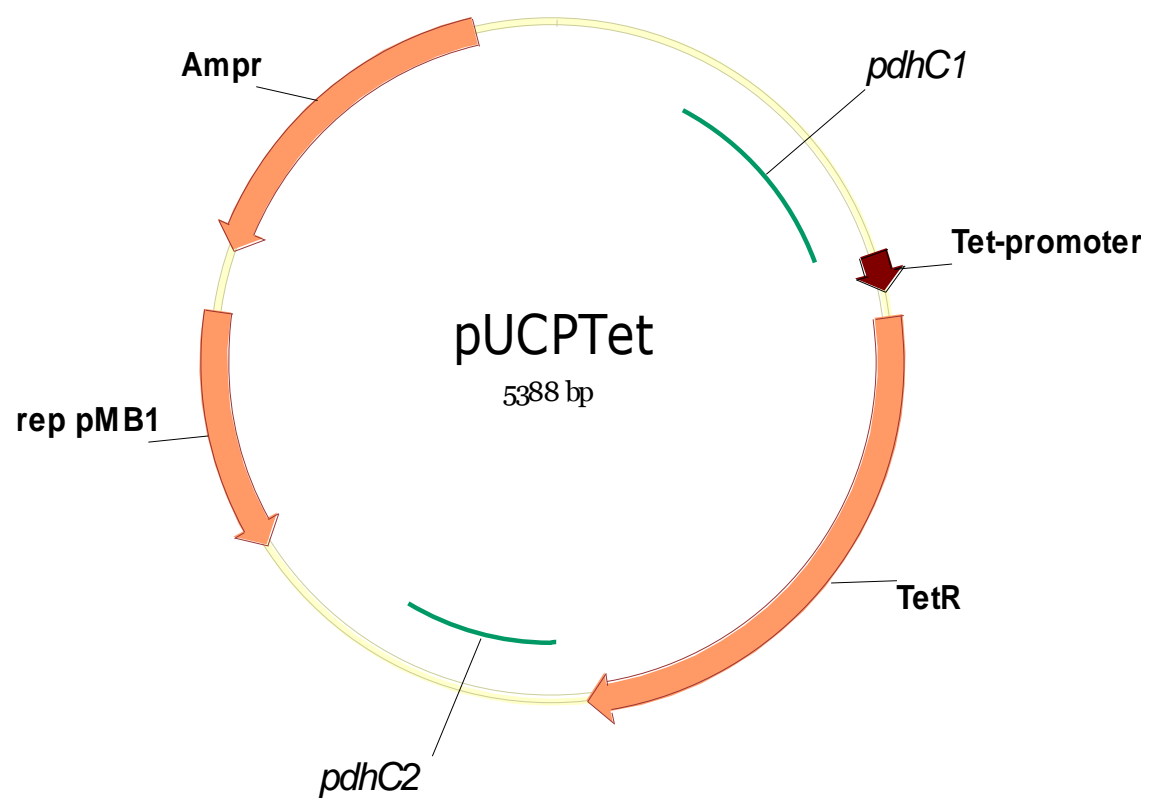**D**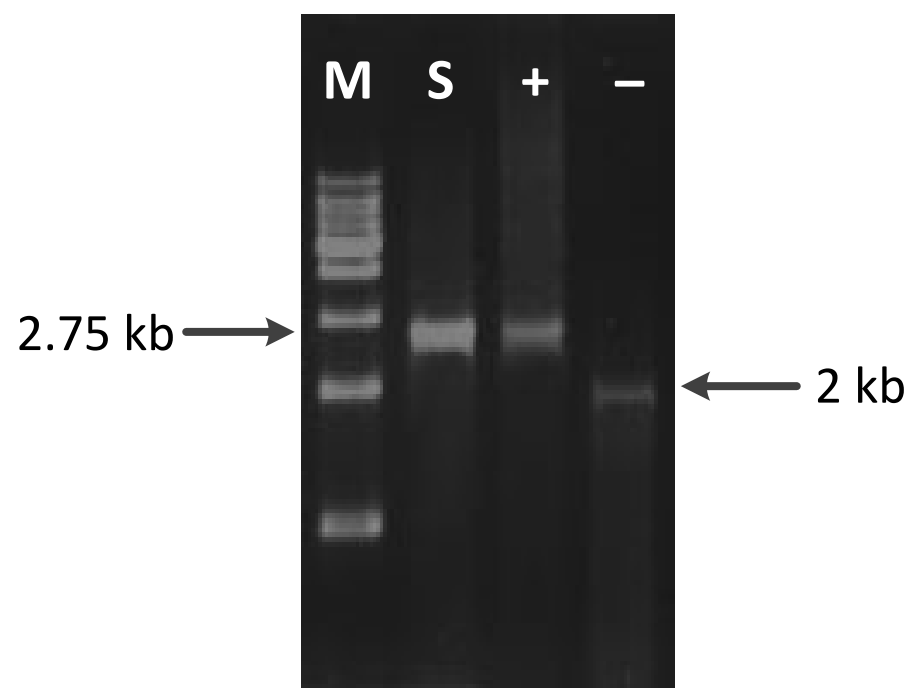

Supplement: Additional file 2 — Plasmids construction and gene knockout confirmation. Plasmids used for gene disruption of ldh (A) and pdhC (C). PCR confirmation of gene knockout for ldh (B) and pdhC (D). The genomic DNA of BSUL03 was used as control. Lane M, 1 kb DNA ladder; Lane S, the positive double crossover mutant; Lane +, the positive control; Lane -, the negative control. [file 1475-2859-11-101-S2.pdf]
