# Supplementary material for: Rational improvement of the engineered isobutanol-producing Bacillus subtilis by elementary mode analysis
Source: Microb Cell Fact. 2012 Aug 3;11:101. doi: 10.1186/1475-2859-11-101 (PMC3475101; doi:10.1186/1475-2859-11-101)
Supplement: Additional file 3 — Oligonucleotides used in this study. [file 1475-2859-11-101-S3.pdf]

Table S3 Oligonucleotides used in this study

Underline stands for the restriction site

Primer name	Sequence (5'→3')
ldh-F	ACGACTCTAGAGAGAAACATACCCTGGAA
ldh-R	CATTTCCCGGGTCATTTAAGTTCAG
*pdhC*1-F	CGGAGAAGCTTGAAGAAGAATACA
*pdhC*1-R	CATTACTGCAGTCTACCTCGTCGTTA
*pdhC*2-F	GGTCGGGATCCCTGCTGACACTGAA
*pdhC*2-R	AGAGAGAATTCATAGCCGCCAGGTC
Tet-F	CGGTGCTGCAGTTGTTGTATAAGTG
Tet-R	ATATAGGATCCCAGGTCGACGGATC
